# Intervention of Gastrodin in Type 2 Diabetes Mellitus and Its Mechanism

**DOI:** 10.3389/fphar.2021.710722

**Published:** 2021-09-16

**Authors:** Yu Bai, Ke Mo, Guirong Wang, Wanling Chen, Wei Zhang, Yibo Guo, Zhirong Sun

**Affiliations:** ^1^ School of Chinese Material Medica, Beijing University of Chinese Medicine, Beijing, China; ^2^ Ningqiang Chinese Herbal Medicine Industry Development Center, Hanzhong, China

**Keywords:** gastrodin, type 2 diabetes, insulin resistance, USP4, AKT pathway

## Abstract

As a severe metabolic disease, type 2 diabetes mellitus (T2DM) has become a serious threat to human health in recent years. Gastrodin, as a primary chemical constituent in Gastrodia elata Blume, has antidiabetic effects. However, the possible mechanisms are unclear. The aim of the present study was to investigate the effects and possible mechanisms of gastrodin on the treatment of T2DM. *In vivo*, after treatment with gastrodin for 6 weeks, fasting blood glucose levels, blood lipid metabolism, and insulin sensitivity index values were remarkably reduced compared with those of the diabetic control group. The values of aspartate aminotransferase and alanine aminotransferase also showed that gastrodin alleviates liver toxicity caused by diabetes. Moreover, gastrodin relieved pathological damage to the pancreas in T2DM rats. *In vitro*, gastrodin alleviated insulin resistance by increasing glucose consumption, glucose uptake, and glycogen content in dexamethasone-induced HepG2 cells. The Western blotting results showed that gastrodin upregulated the expression of insulin receptors and ubiquitin-specific protease 4 (USP4) and increased the phosphorylation of GATA binding protein 1 (GATA1) and protein kinase B (AKT) *in vivo* and *in vitro*. Furthermore, gastrodin decreased the ubiquitin level of the insulin receptor via UPS4 and increased the binding of GATA1 to the USP4 promoter. Additionally, administration of the phosphatidylinositol 3-kinase (PI3K)/AKT signaling pathway inhibitors MK-2206 and LY294002 abolished the beneficial effects of gastrodin. Our results indicate that gastrodin promotes the phosphorylation of GATA1 via the PI3K/AKT pathway, enhances the transcriptional activity of GATA1, and then increases the expression level of USP4, thereby reducing the ubiquitination and degradation of insulin receptors and ultimately improving insulin resistance. Our study provides scientific evidence for the beneficial actions and underlying mechanism of gastrodin in the treatment of T2DM.

## Introduction

Type 2 diabetes mellitus (T2DM) is a metabolic disease with a complex pathogenesis involving systemic disorders of glucose metabolism, amino acid metabolism, and lipid metabolism (Wild et al., 2004). T2DM often causes a high mortality rate and is accompanied by serious complications. T2DM is a heterogeneous disorder characterized by insulin resistance (IR), relative pancreatic beta cell dysfunction, hyperglycemia, dyslipidemia, and uncontrollable glucose and protein metabolism (DeFronzo et al., 2015). Drugs currently available for the treatment of T2DM include biguanide, thiazolidinedione, sulfonylurea, α-glycosidase inhibitors, and insulin (Anyanwu et al., 2019). However, these drugs have several rather large side effects. Metformin is the current first-line drug for the treatment of T2DM and is isolated from extracts of Galega officinalis (Bailey and Day, 2004); its success has motivated the search for other hypoglycemic traditional Chinese medicines (TCMs).

Gastrodin is the main constituent of a traditional Chinese herbal medicine named Gastrodia elata Blume, which is mainly used in the clinical treatment of epilepsy and headache. Gastrodin has a variety of pharmacological activities, such as anti-inflammatory and antioxidative activities blood lipid lowering, and fatty liver and liver fibrosis improvement. It has been reported that gastrodin has hypoglycemic activity and can significantly reduce fasting blood glucose in streptozotocin-induced diabetic mice (Han et al., 2013). Gastrodin has potent activities in lowering blood glucose, improving insulin resistance, and ameliorating diabetic nephropathy (Zhang et al., 2013). Gastrodin intervention ameliorated vascular dysfunction and nitric oxide (NO) signaling deficits in the cerebellum. Gastrodin treatment restored the protein expression of relevant factors and ameliorated neuronal damage (Zhang et al., 2020). Gastrodin reduced neurotoxicity in striatal neurons (Qi et al., 2019). However, the detailed mechanisms of gastrodin in modulating glucose metabolism and increasing insulin sensitivity have not been thoroughly studied.

Some pharmacological effects of gastrodin are related to activation of the Akt signaling pathway. Gastrodin effectively promotes the proliferation of RSC96 Schwann cells (SCs) by activating Akt phosphorylation (Zuo et al., 2016). Activation of the PI3-kinase/AKT signaling pathway induces phosphorylation of GATA-1 by phosphorylating GATA-1S310 and then enhances GATA-1 transcriptional activity (Zhao et al., 2006).

Insulin resistance is a key driver of type 2 diabetes mellitus and is characterized by defective insulin receptor signaling. E3 ubiquitin ligases play pivotal roles in the process of insulin resistance and directly degrades the insulin receptor, insulin receptor substrate, and other key insulin signaling molecules via the ubiquitin-proteasome system (UPS) (Yang et al., 2016). The E3 ubiquitin ligase MARCH1 degrades the cell surface insulin receptor and impairs cellular insulin action (Nagarajan et al., 2016). Ubiquitin-specific protease 4 (USP4), which is a deubiquitinating enzyme, is involved in regulating multiple inflammatory pathways. Hepatocyte-specific USP4 depletion exacerbated hepatic steatosis (HS), IR, and the inflammatory response in high fat diet (HFD)-induced nonalcoholic fatty liver disease (NAFLD) mice (Zhao et al., 2018).

Until now, the mechanisms of gastrodin have not been fully elucidated. Therefore, in this study, we explored the underlying mechanism by which gastrodin improves insulin resistance to learn more about gastrodin and provide scientific evidence for the beneficial actions and traditional use of gastrodin in the treatment of T2DM.

## Materials and Methods

### Chemicals and Reagents

Gastrodin and insulin was purchased from Sigma (St. Louis, MO, United States). Fetal bovine serum (FBS) and Dulbecco’s modified Eagle’s medium (DMEM) were purchased from Gibco (California, CA, United States). 2-NBD-glucose (2-NBDG) was purchased from AAT Bioquest (Sunnyvale, CA, United States).

Other reagents obtained include PVDF membrane (0.45 μm) (Millipore, Schwalbach, Germany); RIPA, cell membrane protein and cytoplasmic protein extraction kit (Beyotime Biotechnology, Shanghai, China); Anti-Na/K ATPase antibody (3,010), Anti-phos-Akt (Ser473) antibody (4,060), Anti-Akt antibody (4,685), Anti-glucagon antibody (2,760), Anti-insulin antibody (3,014) (CST, Boston, United States); Anti-Insulin Receptor antibody (ab206849), Anti-phos-GATA1 (Ser310) antibody (ab181544), Anti-GATA1 antibody (ab194912), Anti-Ubiquitin antibody (ab134953) (Abcam, Cambridge, United Kingdom); Anti-MARCH1 antibody (ABIN2705354) (antibodies-online, Beijing, China); Anti-Insulin Receptor antibody (20433-1-AP) (PROTEINTECH, Rosemont, United States); Anti-USP4 antibody (sc-376000) (Santacruz, United States); iScript cDNA synthesis Kit (Bio-Rad, Hercules, CA, United States); MG-132 (Selleck Chemicals, Houston, Texas, United States); Luciferase Assay Reagent (Promega, Madison, United States); HE staining Kit (Beyotime Biotechnology, Shanghai, China); ALT Kit, AST Kit, TG Kit, TC Kit, HDL-C Kit, LDL-C Kit (Jian Cheng Bioengineering Institute, Nanjing, China); Glucagon Quantikine ELISA Kit, Rat TNF-alpha Quantikine ELISA Kit (R&D Systems, Minnesota, United States).

### Animals

Male Wistar rats (180 ± 10 g, *n* = 36) were obtained from the experimental animal center of Nanjing University. All animals were housed at a constant temperature (23 ± 2°C) with free access to food and water in an specific pathogen free (SPF) animal laboratory. The animal experiment protocol was approved by the Institutional Animal Care and Use Committee of Beijing University of Chinese Medicine.

### Induction of Type 2 Diabetes Mellitus and Experimental Design

Before the study began, all rats were housed for 1 week. Six rats were fed a normal diet as the normal control (NC) group. The other rats were fed a high-fat diet (HFD) (78.8% normal diet, 10% lard oil, 10% egg yolk powder, 1% cholesterol, 0.2% sodium cholate) for 3 weeks (Singh et al., 2017). The animals were fasted for 12 h and received an intraperitoneal injection of STZ (35 mg/kg, 0.1 M citric acid/sodium citrate buffer) (Srinivasan et al., 2005). After 72 h, rats were fasted for 12 h, and the fasting blood glucose (FBG) levels of the rats were determined. The rats with an FBG of >11.1 mmol/L were used as T2DM animals.

T2DM animals were divided randomly into five groups: the diabetic control (DC) group, gastrodin-L group (30 mg/kg), gastrodin-M group (60 mg/kg), gastrodin-H group (90 mg/kg), and metformin group (250 mg/kg). The T2DM rats were given gastrodin or metformin solution delivered into the stomach by a gavage probe once a day for 6 weeks. The NC and DC groups were given the same volume of normal saline once a day for 6 weeks.

### Blood Glucose Levels, Oral Glucose Tolerance Test, and Insulin Tolerance Test

All rats were fasted for 12 h. The end of the mouse tail was cut off by 1–2 mm with scissors, and the tail was gently squeezed to collect the blood into a drop. Then the blood glucose levels were measured using a glucometer (Accu-Chek Guide, Roche, Switzerland) 2 h after the last administration of gastrodin.

For the OGTT, after 6 weeks of intragastric administration of gastrodin, the 12-h-fasted rats in all groups were given aqueous glucose solution (2.0 g/kg) delivered into the stomach by a gavage probe. Blood glucose levels were determined at 0, 15, 30, 60, and 120 min after glucose administration.

For the ITT, mice were intraperitoneally injected with recombinant human insulin (1 IU/kg) 48 h after the end of the OGTT. Blood glucose levels were measured at 0, 15, 30, 60, and 120 min after the injection of insulin.

### Fasting Serum Insulin Levels and Insulin Sensitivity Index

Insulin levels were measured by using ELISA in blood samples 2 h after the last administration of gastrodin. The insulin sensitivity index (ISI) was calculated according to the formula: ISI = 1/[blood glucose (mM/L) × blood insulin (μIU/ml)] (Wang et al., 2013).

### Biochemical Analysis

After 6 weeks of treatment with gastrodin and metformin, the rats were sacrificed, and blood samples were obtained from the orbital sinus and centrifuged (3,000 rpm for 10 min) to isolate the serum. Fasting serum insulin (FINS) concentrations were measured using an enzyme-linked immunosorbent assay (ELISA) kit according to the manufacturer’s instructions (R&D Systems, Minnesota, United States). Total cholesterol (TC), triglyceride (TG), low-density lipoprotein cholesterol (LDL-c), and high-density lipoprotein cholesterol (HDL-c) were measured using a biochemical analyzer (Jian Cheng Bioengineering Institute, Nanjing, China). Alanine aminotransferase (ALT) and aspartate aminotransferase (AST) in serum were measured using an automatic multiscan spectrum (LB941, Berthold Technologies, Germany).

### Histopathology

At the end of the experiment, the liver and pancreatic tissues were removed and fixed in formalin solution. Then, the samples were dehydrated, embedded in paraffin, and prepared for observation under a light microscope by slicing to a thickness of 4 μm and staining with hematoxylin-eosin (HE).

Immunohistochemical staining of insulin and glucagon was performed as described previously with slight modifications (Jiang et al., 2015). Pancreatic tissue sections were first dehydrated in xylene, hydrated with ethanol, and then rinsed with distilled water. Next, the sections were placed in a 3% hydrogen peroxide solution for 10 min to block endogenous peroxide activity. Subsequently, different pancreatic tissue sections from the same rat were incubated overnight at 4°C with anti-insulin antibody and anti-glucagon antibody. After washing 3 times with PBS, a secondary antibody was added to the sectioned tissue and incubated at 37°C for 1 h. Finally, pancreatic tissue sections were exposed to diaminobenzidine (DAB), developed under a microscope, and counterstained with Meyer hematoxylin.

### Cell Culture, Induction of IR-HepG2 and Screening of Concentrations of Gastrodin

HepG2 cells were cultured in DMEM supplemented with antibiotics (100 U/mL penicillin A and 100 U/mL streptomycin) and 10% fetal bovine serum and were maintained at 37°C in a humidified incubator containing 5% CO_2_. Dexamethasone (Dex) was used to induce insulin resistance. After cells reached 70–80% confluence, the cells were then cultured in DMEM supplemented with 1 μM Dex in the absence or presence of different concentrations of gastrodin (10, 30, 100, 300, and 1,000 μM) for 24 h. Then, cell viability was determined by using a CCK8 kit to screen the appropriate concentration of gastrodin treatment.

### Glucose Uptake Assay

Cells were seeded into a 96-well plate. After 24 h, the medium was replaced with KRB solution for 2 h, and the cells were then incubated with 100 μM insulin and gastrodin for 24 h. The standard substance (100 pmol) was diluted into different concentrations of standard solutions (2, 4, 6, 8, and 10 pmol/μL) with sample diluent, and 100 μL standard solution was added to each well. Next, cells were exposed to 150 ng/ml 2-NBDG for another 30 min. Then, the supernatant was discarded and washed with KRB, and 2-NBDG fluorescence intensity was measured with a fluorescence microplate reader (Berthold LB941, Germany) at an excitation wavelength of 485 nm and emission wavelength of 540 nm. After detecting the fluorescence intensity, the cells were digested with trypsin, resuspended with PBS, and counted by flow cytometry. The number of cells in the normal control group was taken as 1, and the relative cell numbers of the other groups were obtained by comparing with the normal control group. The corresponding fluorescence intensity was normalized by number of cells, and the 2-NBDG uptake data was the 2-DG uptake per 10^5^ cells.

### Glycogen Content Assay

HepG2 cells were seeded into 24-well cell culture plates. Following 24 h of stabilization, the cells were incubated with different concentrations of gastrodin and 100 μM insulin for 24 h. Glycogen content was measured by using a glycogen assay kit. The protein content was quantified by using a BCA Protein Assay Kit. Glycogen content was normalized to protein level, and values are presented as the ratio of glycogen to protein.

### Western Blot

After pretreatment, cells were washed with ice-cold PBS, lysed with lysis buffer, and centrifuged at 12,000 rpm for 5 min at 4°C. The protein concentration was determined with BCA Protein Assay Reagent. Total proteins (30–50 μg) were separated by SDS-PAGE and then transferred to PVDF membranes. Membranes were blocked in a blocking buffer composed of 5% skim milk powder dissolved in Tris-buffered saline containing 0.1% Tween 20 (TBST) for 1 h at room temperature. Subsequently, blots were washed and incubated overnight at 4°C with primary antibody. The membranes were incubated with secondary antibody for 1 h at room temperature after three washes with TBST. After the chemiluminescence reaction, bands were detected, and densities were evaluated using Image-Pro Plus 6.0 software and normalized for β-actin density for quantitative analysis.

### Real-Time PCR Analysis

Total RNA was isolated from cells with TRIzol reagent according to the manufacturer’s instructions. β-actin was used as an internal control. Real-time PCR was performed on an ABI 7500 System (Applied Biosystems, United States) by using the SYBR Premix Ex Taq™ PCR kit (TaKaRa, Dalian, China). The reverse transcription reaction conditions were as follows: 25°C for 5 min, 42°C for 30 min, and 85°C for 5 min. Real-time PCR data were analyzed using the 2^−ΔΔCT^ method with the SDS Software package (Applied Biosystems). PCR primers were shown in Table 1.

**TABLE 1:** Primers used in Quantitative Real-Time PCR.

Primers	Sequence (5’→3’)	
Insulin Receptor	Forward	5’-CTG​TCA​CCG​GGG​AAC​TAC​AG -3’
	Reverse	5’-ACG​TAG​AAA​TAG​GTG​GGT​TCC​G -3’
β-actin (human)	Forward	5’- CAC​CAT​TGG​CAA​TGA​GCG​GTT​C -3’
	Reverse	5’-AGG​TCT​TTG​CGG​ATG​TCC​ACG​T -3
β-actin (Rat)	Forward	5’-ATC​TGG​CAC​CAC​ACC​TTC -3
	Reverse	5’-AGC​CAG​GTC​CAG​ACG​CA -3
USP4-qpcr	Forward	CCT​CCA​AGA​GGA​GGG​TTG​TG
	Reverse	CGC​AGA​ATT​CCG​TCA​GCT​CT

### Coimmunoprecipitation (Co-IP) Assay

The Co-IP assay was used to assess the ubiquitin level of insulin receptors and the interactions of USP4, MARCH1, and insulin receptors. HepG2 cells were lysed by RIPA lysis. The cell lysates were incubated with anti-ubiquitin antibody, anti-USP4 antibody, anti-MARCH1 antibody, and Protein A agarose beads. Then, the protein level of insulin receptor in the immunoprecipitation complex was detected by Western blot.

### Chromatin Immunoprecipitation (CHIP) Assay

The ChIP assay was performed by using the Magna ChIP™ G Tissue Kit (Magna, U.S.A.). In brief, HepG2 cells were fixed with formaldehyde for 10 min at 37°C, followed by incubation for 10 min with SDS lysis buffer. Cells were lysed with ChIP lysis buffer and sonicated later. The 10 μL cell lysate was stored at −80°C as input, and the remaining cell lysate was incubated with anti-GATA1 at 4°C for 24 h. Then, Protein A/G magnetic beads were added to lysates to prepare the immunoprecipitated protein-DNA complexes. The full-length promoter sequence of the pre-selected target gene USP4 was searched, and the action sites of USP4 and GATA1 were found by combining with the target gene prediction software. Primer 5 was used to design primers so that the PCR product contained the action site (AACTAATGTGG) of binding site 1 of GATA1 and the USP4 promoter. DNA was extracted from complexes, and the expression of USP4 in DNA samples was detected by qPCR.

### Statistical Analysis

Statistical significance between the data from different groups was determined by one-way analysis of variance (ANOVA) followed by Dunnett’s post hoc test. Statistical analyses were performed with GraphPad Prism 8.0.

## Results

### Gastrodin Reduced Blood Glucose and Improved Oral Glucose Tolerance and Insulin Sensitivity Index (ISI) of Type 2 Diabetes Mellitus in Rats

**FIGURE 1:**
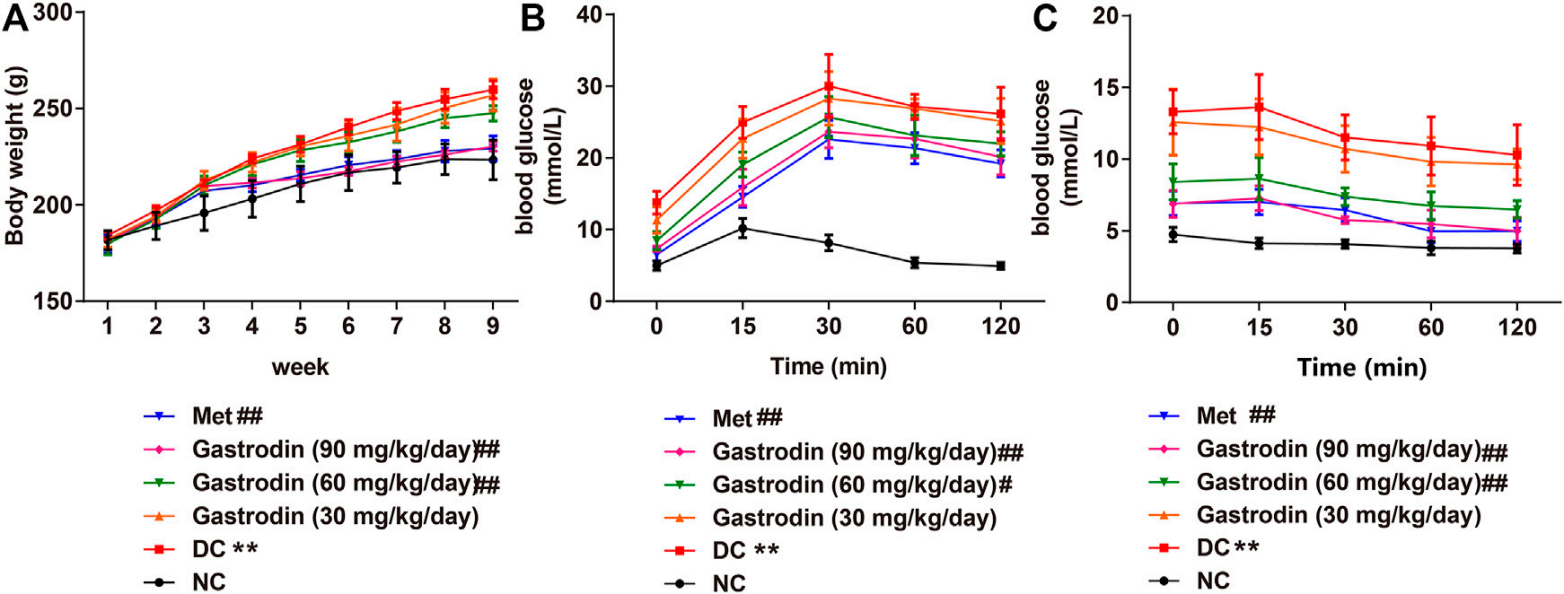
The effects of Gastrodin on body weight, OGTT and ITT in T2DM rats. Body weight **(A)**, oral glucose tolerance test curve of each group **(B)** and insulin tolerance test curve of each group of each group **(C)**. The results were presented as mean ± SD (*n* = 6). **p < 0.01, vs. NC group, #*p* < 0.05, ##*p* < 0.01, vs. DC group.

We observed a significant increase in the body weight of T2DM rats (*p* < 0.01) over the experiment (Figure 1A). However, the rats treated with metformin, gastrodin-M, and gastrodin-H effectively prevented the body weight increase (*p* < 0.01). To analyze oral glucose tolerance (OGTT), the glucose tolerance test was performed for each mouse in different groups. As shown in Figure 1B, the outcomes of the OGTT in the gastrodin and Met groups were improved to some extent. The utilization of glucose in the gastrodin-M and gastrodin-H groups were significantly improved compared with the DC group (*p* < 0.05, *p* < 0.01). In the insulin tolerance test, blood glucose levels in all groups decreased after insulin injection, but the blood glucose level of the DC group was still significantly higher than that of the NC group (Figure 1C). Compared with the DC group, the blood glucose levels in the metformin group and the gastrodin-M and gastrodin-H groups were significantly decreased.

**FIGURE 2:**
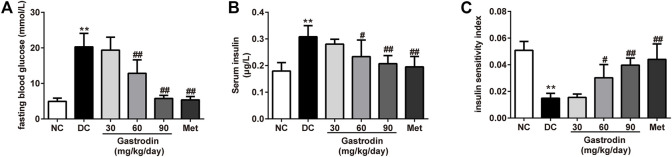
The effects of Gastrodin on blood glucose levels, FINS and insulin sensitivity index in T2DM rats. Fasting blood glucose level **(A)**, Serum insulin concentration **(B)** and insulin sensitivity index (ISI) of each group **(C)**. The results were presented as mean ± SD (*n* = 6). **p < 0.01, vs. NC group, #*p* < 0.05, ##*p* < 0.01, vs. DC group.

Figure 2A shows the FBG level of each group. Compared with the NC group, the FBG in the DC group increased significantly. After oral administration of gastrodin for 6 weeks, compared to the DC group, the FBG in the gastrodin-M and gastrodin-H groups showed 36.73% (*p* < 0.01) and 71.65% (*p* < 0.01) decreases in FBG levels, respectively. As shown in Figure 2B, the FINS level of the DC group (0.308 ± 0.042 μg/L) was significantly higher than that of the NC group (0.180 ± 0.031 μg/L) (*p* < 0.01). However, after 6 weeks of treatment, compared to the model group, the FINS of both the gastrodin-M (0.234 ± 0.062 μg/L, *p* < 0.05) and gastrodin-H (0.207 ± 0.030 μg/L, *p* < 0.01) groups was significantly reduced. The FINS level significantly decreased in the metformin group (0.195 ± 0.039 μg/L, *p* < 0.01). Additionally, Figure 2C shows that the insulin sensitivity index (ISI) was predominantly decreased in the model group compared with the NC group (*p* < 0.01). The ISI values of the gastrodin-M and gastrodin-H groups were significantly higher than those of the DC group, implying that sensitivity to insulin could be enhanced and insulin resistance could be reduced by gastrodin.

### Antihyperlipidemic Effects of Gastrodin

**FIGURE 3:**
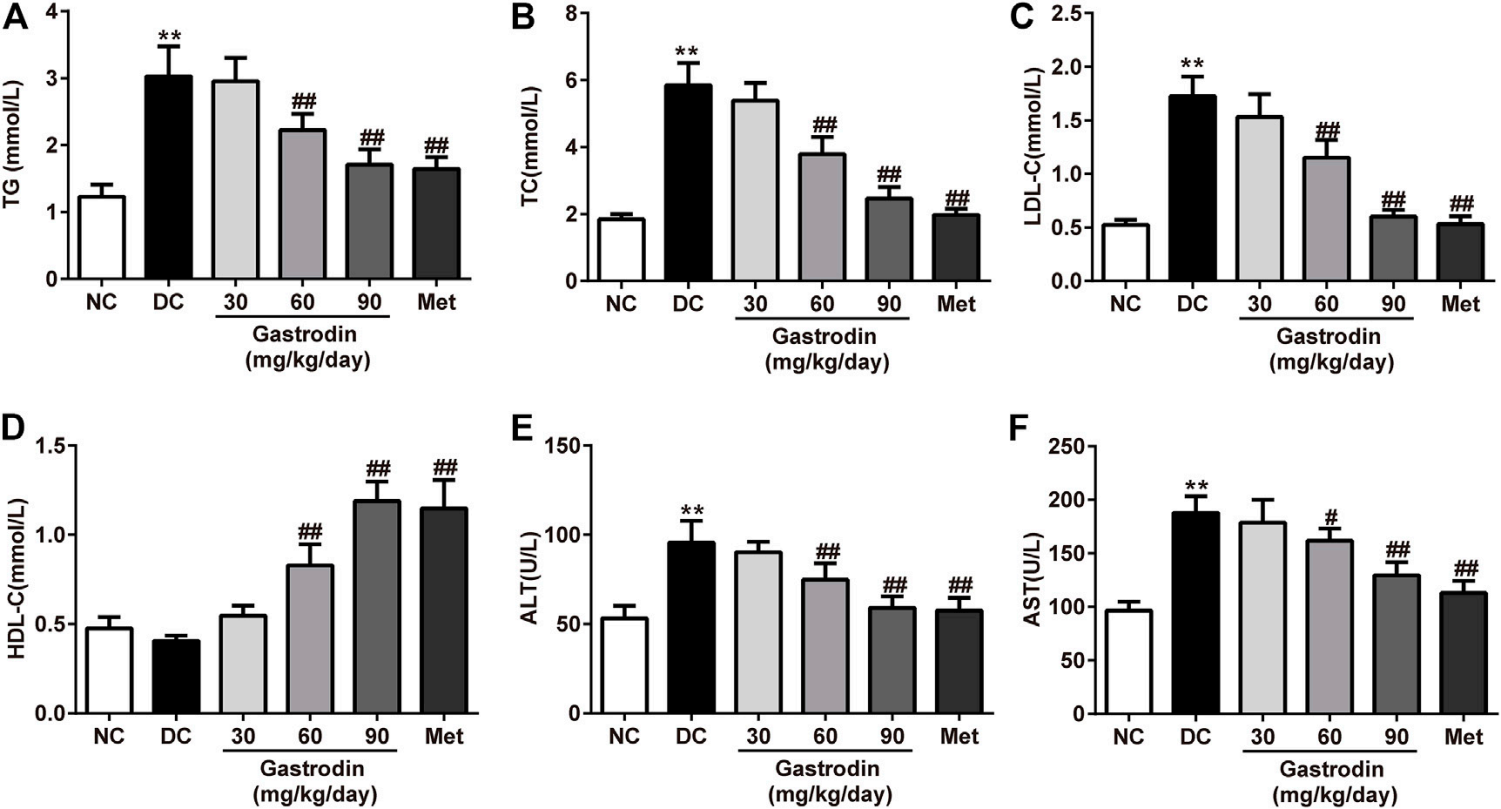
The effects of Gastrodin on serum lipid profiles. Effects of Gastrodin on the TG **(A)**, TC **(B)**, LDL cholesterol **(C)**, HDLcholesterol **(D)**, ALT and AST levels in T2DM rats. The results were presented as mean ± SD (*n* = 6). **p < 0.01, vs. NC group, #*p* < 0.05, ##*p* < 0.01, vs. DC group.

Figures 3A–D shows the lipid profiles of each group. At the end of the study, the TC, TG, and LDL-c levels of the DC group significantly increased (*p* < 0.01), and HDL-c levels of the DC group decreased. However, after 6 weeks of treatment, the levels of TC, TG, and LDL-c in the gastrodin-M and gastrodin-H groups (*p* < 0.01) significantly decreased, and HDL-c levels significantly increased compared to those in the DC group. These findings were similar to those of the metformin group, which provides clear evidence of an antihyperlipidemic effect. Compared with the normal control group, AST and ALT levels increased significantly in the model group (*p* < 0.01) and decreased significantly after interventions (*p* < 0.01, Figures 3E,F). The results indicated that metformin modified dyslipidemia with a similar trend to gastrodin. In addition, the values of AST and ALT also showed that gastrodin alleviates liver toxicity caused by diabetes.

### Histopathological Changes in Pancreas Tissues

**FIGURE 4:**
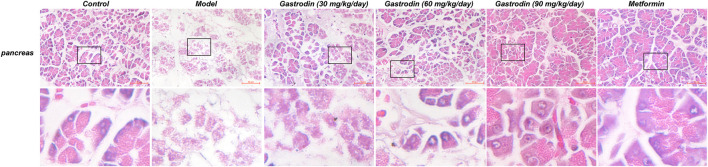
HE staining of the pancreas in rat. (*n* = 6).

Figure 4 shows the histological pancreatic tissue excised from experimental rats under ×400 magnification. The pancreatic islets of the NC group were round and cord-like, and the edges were neat and of normal size. Hematoxylin-eosin (HE) staining of islet cells was lightly colored and showed no obvious degeneration. However, the islet cells of the DC group were irregular in shape and unevenly distributed, showing significant decreases in size and number. The islet cells were moderately degenerated. After drug application, the degree of degeneration and atrophy of the islets in each drug-administered group was alleviated.

**FIGURE 5:**
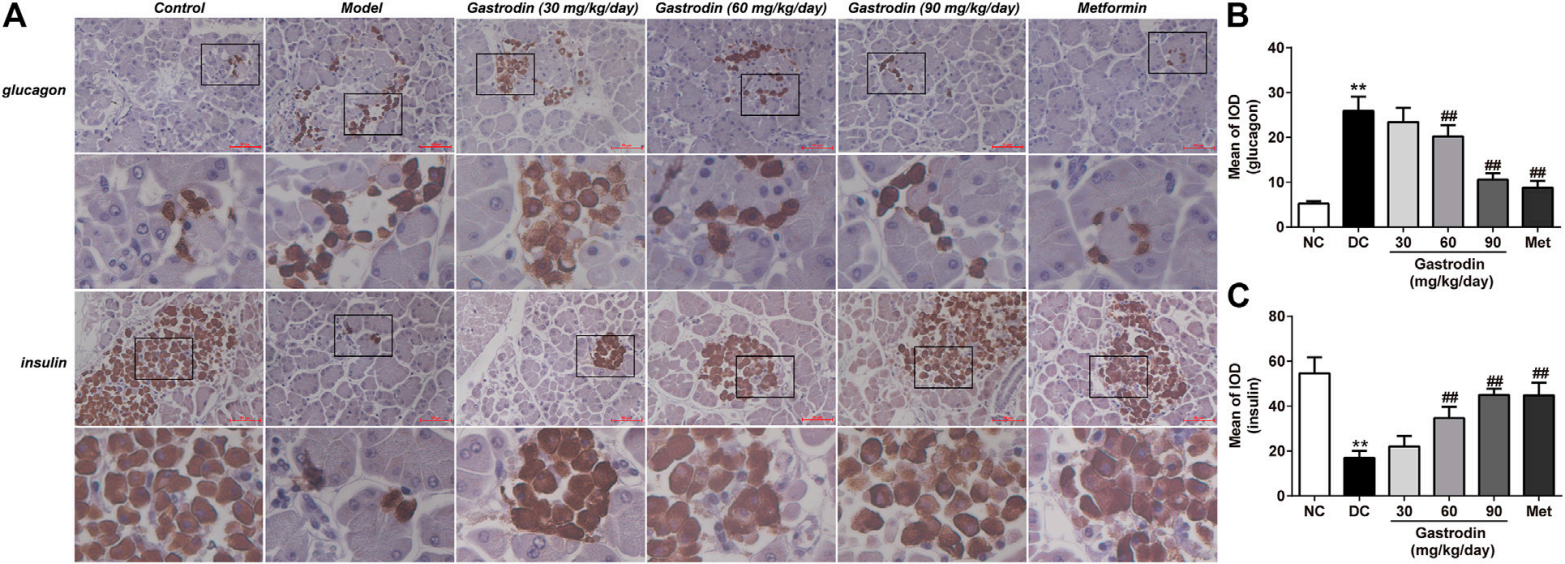
Glucagon and insulin staining of the pancreas tissues in rat. The results were presented as mean ± SD (*n* = 6). **p < 0.01, vs. NC group, #*p* < 0.05, ##*p* < 0.01, vs. DC group.

Prompted by the above observations in the pancreas, we subsequently performed insulin and glucagon immunostaining of pancreatic sections to directly examine the curative effect by improving the expression of B-cells (Figure 5A). Immunostaining for glucagon and insulin was quantitated by calculating the ratios of integrated optical density (IOD) to area of interest (AOI) (IOD/AOI) to evaluate the intensities of positive staining (Figures 5B,C). These calculations revealed that T2DM rats showed more positive A-cell (*p* < 0.01) expression and less positive B-cell expression (*p* < 0.01) than healthy controls. However, gastrodin-M and gastrodin-H treatment significantly reduced the number of islet A-cells (*p* < 0.01) and increased islet B-cell expression (*p* < 0.01).

### Gastrodin Upregulated Akt Phosphorylation Levels and the Expression of USP4 in the Livers of Rats

**FIGURE 6:**
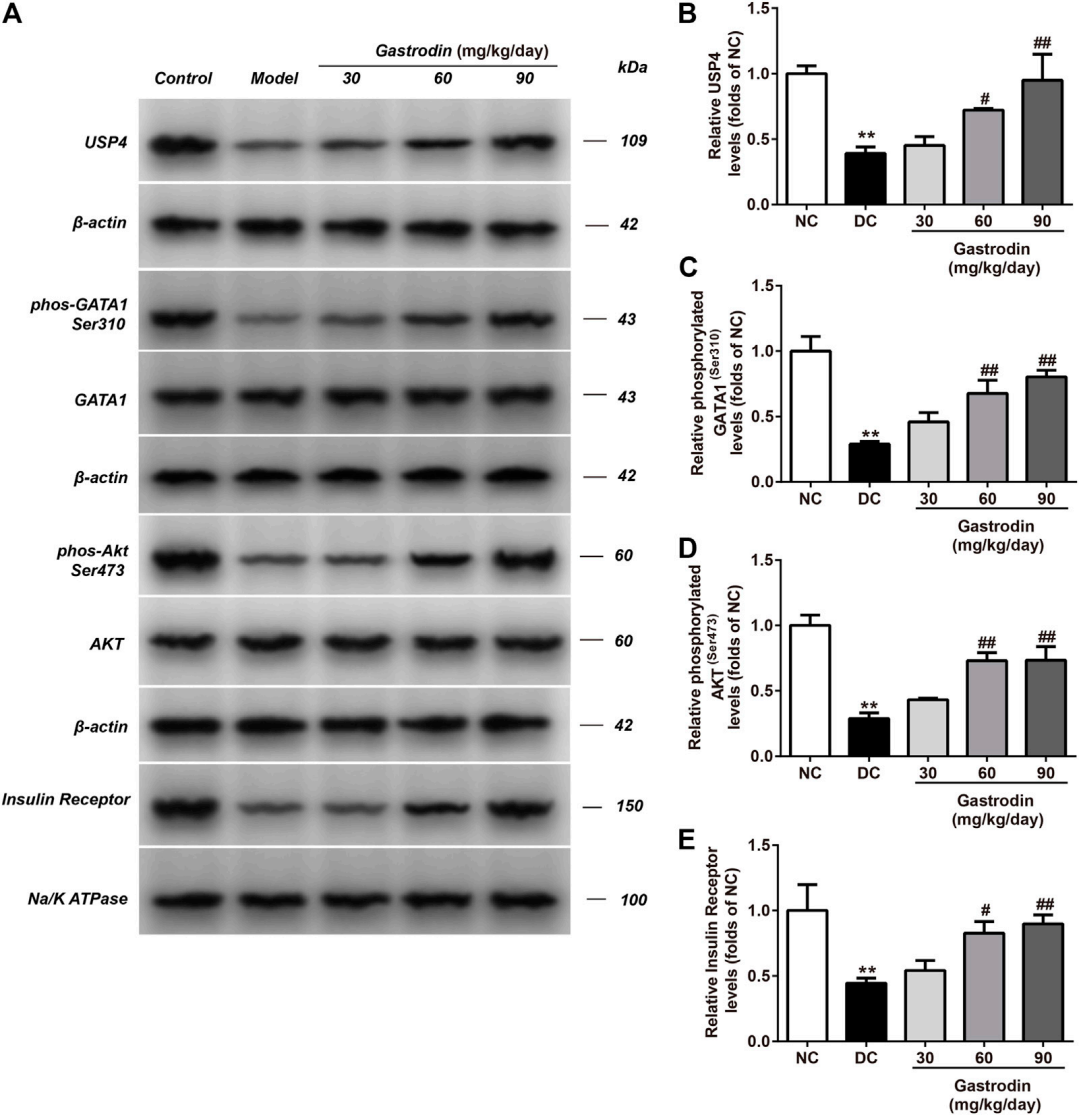
The phosphorylation levels of AKT, GATA1 and the levels of USP4, insulin receptor expressions in liver from different groups were detected by western blot assay, and representative bands were shown in **(A)**. The phosphorylation levels of GATA1 **(C)**, AKT **(D)** and the levels of USP4 **(B)**, insulin receptor **(E)** were normalized to NC. The results were presented as mean ± SD (*n* = 3). **p < 0.01, vs. NC group, ##*p* < 0.01, vs. DC group.

Figure 6 shows the alterations in the *p*-Akt/total Akt ratio and USP4 in the livers of rats. A significant decrease in the *p*-Akt/total Akt ratio and USP4 expression was observed in the IR group compared to the normal control group. The ratio of *p*-Akt/total Akt and USP4 expression in the IR group decreased by 71.24 and 60.90% in the liver (Figures 6B,D), respectively. Compared to the IR group, gastrodin upregulated the ratio of *p*-Akt/total Akt by 50.09, 153.81, and 155.27% and upregulated USP4 expression by 15.66, 84.50, and 143.24% at 10, 30, and 100 μg/ml, respectively.

### Gastrodin Enhanced 2-NBDG Uptake and Increased Glycogen Content in HepG2 Cells

**FIGURE 7:**
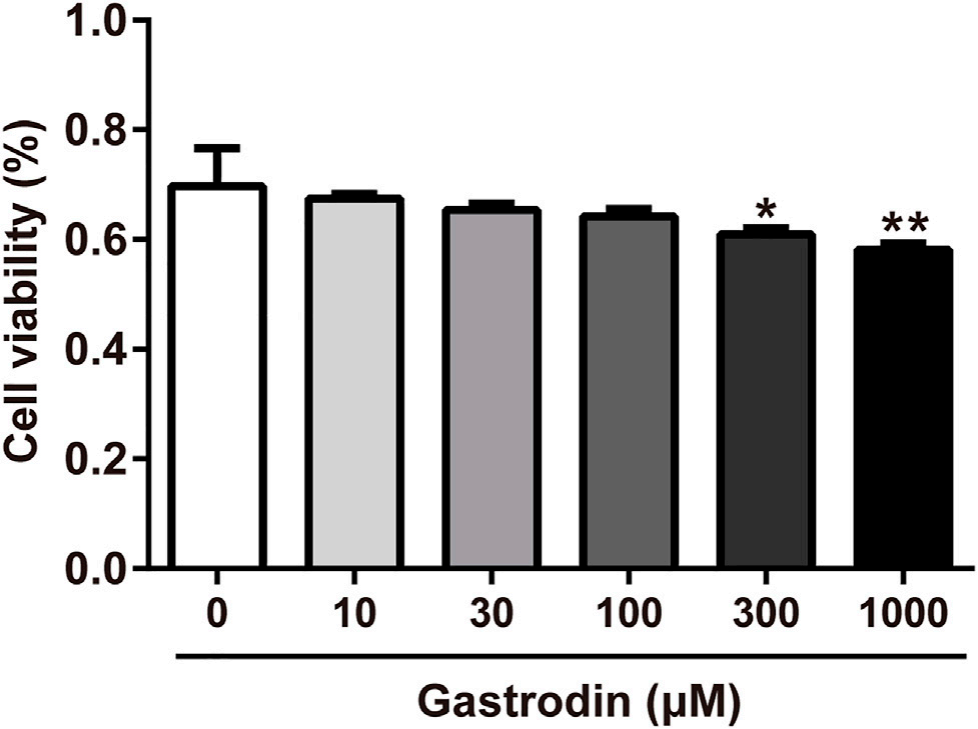
Inhibitory effect of Gastrodin in HepG2 cells. Results were expressed as mean ± SD (*n* = 3). *p < 0.05, **p < 0.01 vs Control group.

As shown in Figure 7, gastrodin treatments (300 and 1,000 μM) significantly inhibited HepG2 cell viability compared with the control group. Therefore, gastrodin concentrations of 10, 30, and 100 μM were selected for subsequent experiments.

**FIGURE 8:**
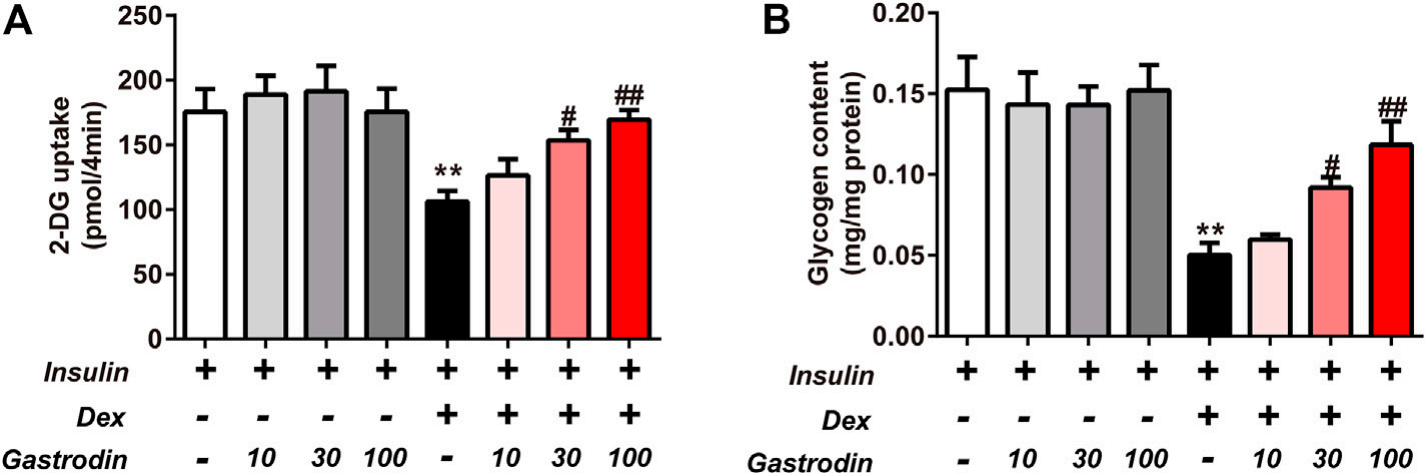
Effects of Gastrodin on Glucose uptake assay and Glycogen content assay in HepG2 cells. The fluorescence intensity of 2-NBDG into HepG2 cells **(A)**. The glycogen content in HepG2 cells **(B)**. The results were presented as mean ± SD (*n* = 3). **p < 0.01, vs. insulin group, #*p* < 0.05, ##*p* < 0.01, vs. Insulin + Dex group.

The glycogen concentration and the fluorescence intensity of 2-NBDG are presented in Figure 8. Compared to the normal control cells, an obvious decrease in 2-NBDG uptake was exhibited in IR model cells (39.54%), indicating that IR-treated cells were successfully induced. However, the uptake of HepG2 cells was enhanced in each gastrodin treatment group. The 2-DG uptake was significantly increased by 44.56 and 59.65% in the 30 and 100 μg/ml gastrodin groups, respectively (*p* < 0.05) (Figure 8A). Gastrodin (30 and 100 μg/ml) showed a significant protective effect on 2-NBDG uptake. As shown in Figure 8B, Dex treatment significantly decreased glycogen content in the IR group. Compared to the IR group, we observed significantly increased glycogen content in HepG2 cells after treatment with 30 and 100 μg/ml gastrodin.

### Gastrodin Enhanced Insulin Receptor Expression and Decreased the Ubiquitin Level of the Insulin Receptor via USP4 in HepG2 Cells

**FIGURE 9:**
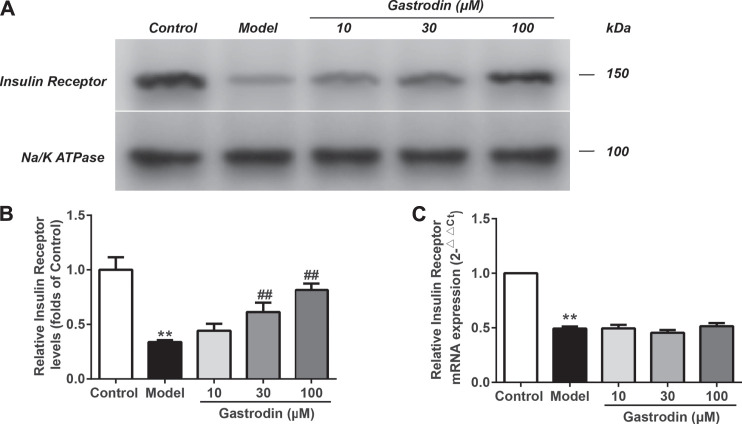
The levels of insulin receptor expressions and mRNA expressions of insulin receptor in HepG2 from different groups. The representative bands were shown in **(A)**. The levels of insulin receptor **(B)** were normalized to control. The mRNA levels of insulin receptor **(C)** was normalized to control. The results were presented as mean ± SD (*n* = 3). **p < 0.01, vs. control group, ##*p* < 0.01, vs. Model group.

As shown in Figures 9A,B, compared with the normal control group, the levels of insulin receptor expression in the model group significantly decreased. In addition, the levels of insulin receptor expression were significantly increased in the gastrodin (30 and 100 μM) groups compared with the model group. As shown in Figure 9C, the mRNA expression of insulin receptors in the model group significantly decreased compared with that in the control group, while the gastrodin groups had no significant effect on the mRNA expression of insulin receptors. These results indicated that gastrodin improved insulin resistance by decreasing insulin receptor degradation rather than upregulating its transcriptional activity.

**FIGURE 10:**
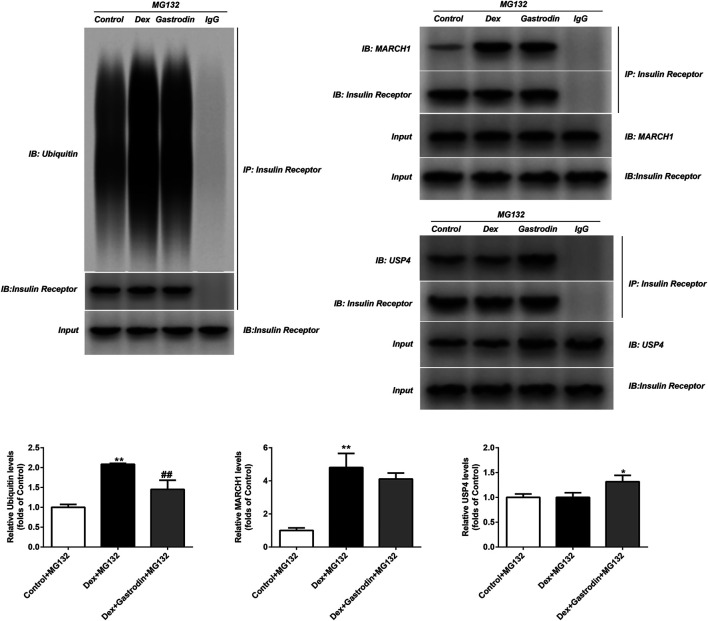
The ubiquitin level of insulin receptor and levels of MARCH1, USP4 and insulin receptor were measured with Co-IP (*n* = 3). MG132 was a proteasome inhibitor. The concentration of gastrodin was 100 μM. *p < 0.05, **p < 0.01, vs. control+MG132 group, ##*p* < 0.01, vs. Dex+MG132 group.

As shown in Figure 10, dexamethasone increased the ubiquitin level of the insulin receptor in the model group compared with the control group, and gastrodin reversed this effect. The binding of MARCH1 and insulin receptor increased in the model group compared with the control group, but gastrodin had no effect on it. Dexamethasone did not affect the binding of USP4 to insulin receptors compared with the control group, but the expression of USP4 and the binding of USP4 to insulin receptors increased after the addition of gastrodin. MG132 was added to avoid the degradation of insulin receptors by proteasomes after ubiquitination, thus avoiding false negative or false positive results. These results suggested that USP4 could be involved in the decrease in ubiquitin levels of insulin receptors mediated by gastrodin.

### Gastrodin Upregulated the Expression of USP4 in HepG2 Cells

**FIGURE 11:**
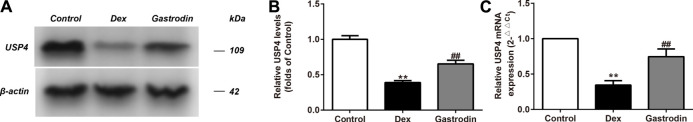
The levels of USP4 expressions and mRNA expressions of USP4 in HepG2 from different groups. The representative bands were shown in **(A)**. The levels of USP4 **(B)** were normalized to Dex. The mRNA levels of USP4 **(C)** was normalized to Dex. The concentration of gastrodin was 100 μM. The results were presented as mean ± SD (*n* = 3). **p < 0.01, vs. Control group, ##*p* < 0.01, vs. Dex group.

Compared with the control group, the levels of USP4 expression significantly decreased in the model group, and the levels of USP4 expression significantly increased in the gastrodin group compared with the model group (Figures 11A,B). As shown in Figure 11C, the mRNA expression level of USP4 significantly decreased in the model group compared with the control group. The mRNA expression level of USP4 was significantly increased in the gastrodin group compared with the model group.

### Gastrodin Stimulated the PI3K/AKT Pathway and Increased the Levels of USP4 Expression by Enhancing the Phosphorylation of GATA1 in HepG2 Cells

**FIGURE 12:**
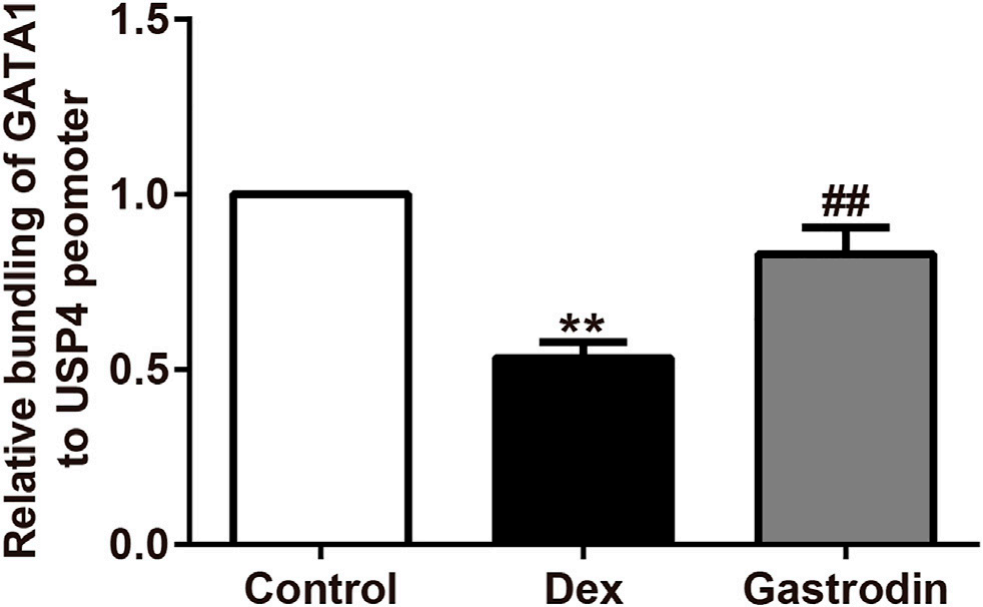
Chromatin immunoprecipitation (ChIP) assay on the promoter of GATA1. The results were presented as mean ± SD (*n* = 3). **p < 0.01, vs. Control, ##*p* < 0.01, vs. Dex. The concentration of gastrodin was 100 μM.

**FIGURE 13:**
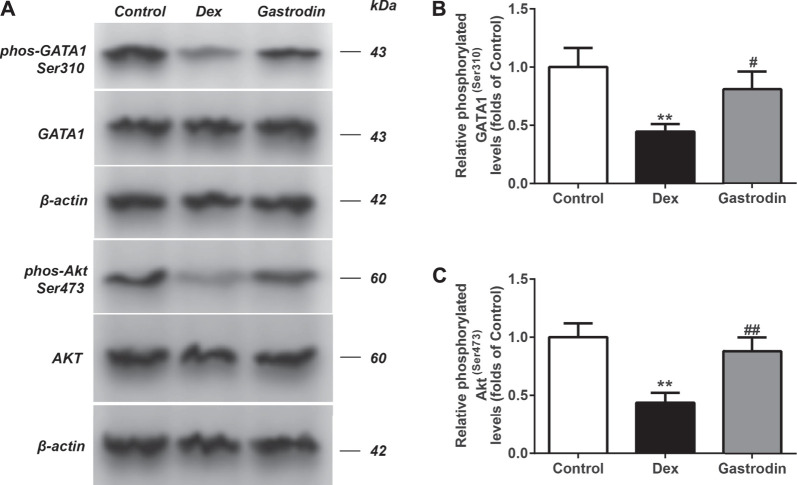
The phosphorylation levels of AKT, GATA1 in HepG2 from different groups were detected by western blot assay, and representative bands were shown in **(A)**. The phosphorylation levels of GATA1 **(B)** and AKT **(C)** were normalized to Dex. The results were presented as mean ± SD (*n* = 3). **p < 0.01, vs. Control, ##*p* < 0.01, vs. Dex.

**FIGURE 14:**
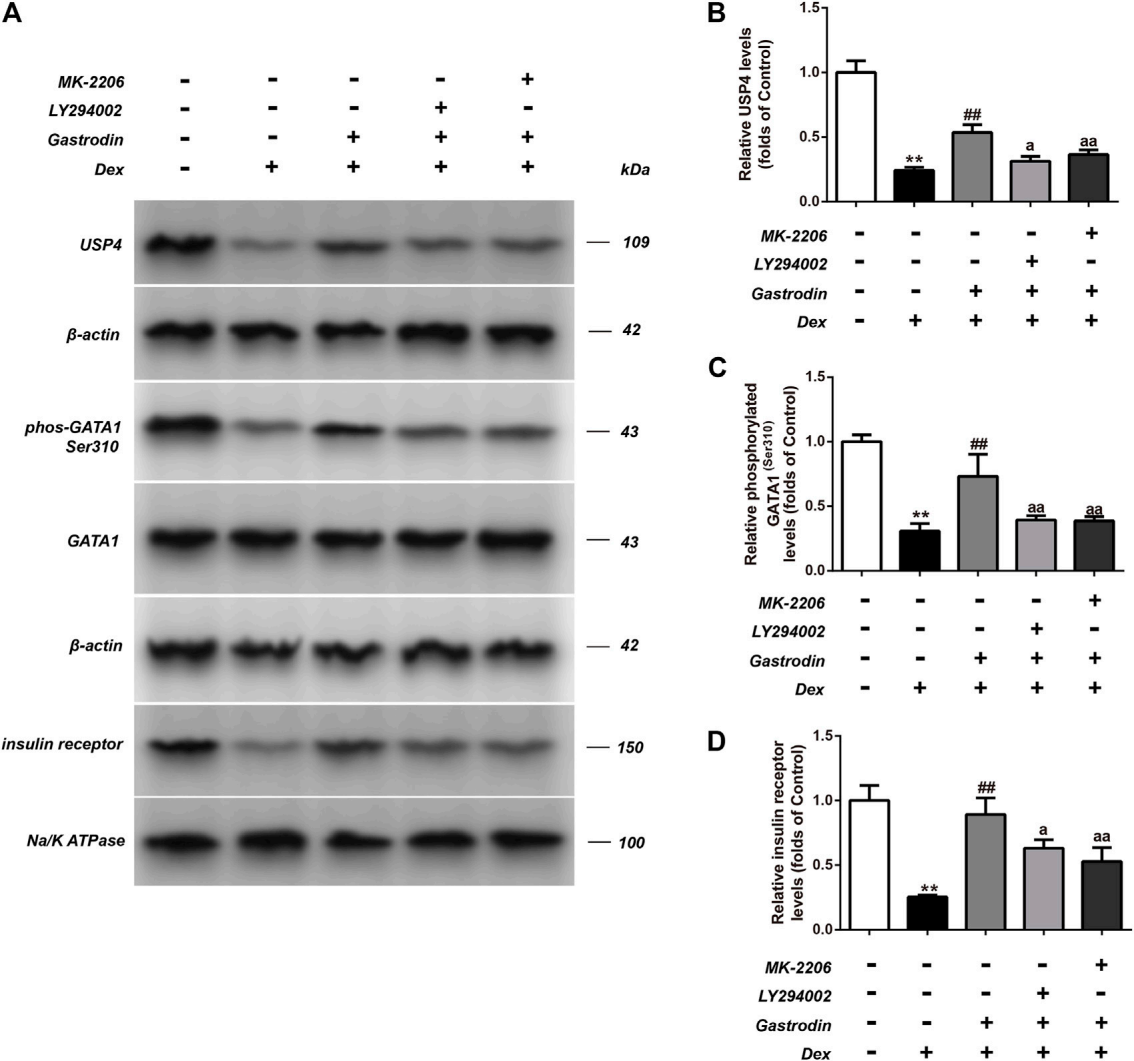
The phosphorylation levels of GATA1 and the levels of USP4 expressions in HepG2 from different groups were detected by western blot assay, and representative bands were shown in **(A)**. The phosphorylation levels of GATA1 **(C)**, the levels of USP4 **(B)** and the levels of insulin receptor **(D)** were normalized to Dex. The results were presented as mean ± SD (*n* = 3). **p < 0.01, vs. Control, ##*p* < 0.01, vs. Dex, ^a^p < 0.05, ^aa^p < 0.01, vs. Dex + Gastrodin group.

Figure 12 shows that compared with the control group, the binding level of GATA1 and the USP4 promoter was significantly decreased in the model group, whereas the binding level of GATA1 and the USP4 promoter was significantly increased in the gastrodin group compared with the model group. As shown in Figure 13, western blot analysis revealed that the expression levels of phospho-GATA1 and phospho-Akt in HepG2 cells were significantly reduced in the model group compared with the control group, while 100 μg/ml gastrodin for 24 h significantly increased the expression of phospho-GATA1 and phospho-Akt in HepG2 cells. Furthermore, the phosphorylation level of GATA1 and the level of USP4 expression were significantly increased in gastrodin group compared with the model group, but the PI3K inhibitor MK-2206 and AKT inhibitor LY294002 both significantly suppressed the effects of gastrodin on the phosphorylation level of GATA1 and the level of USP4 expression in HepG2 cells (Figure 14). Therefore, these results suggest that gastrodin stimulates the PI3K/AKT pathway and that activation of the PI3K/AKT pathway promotes USP4 expression by enhancing the phosphorylation of GATA1 in HepG2 cells.

## Discussion

T2DM is mainly characterized by a progressive decline in insulin action (insulin resistance), followed by the inability of beta cells to compensate for insulin resistance (pancreatic beta cell dysfunction) (Lebovitz and Banerji, 2004). T2DM rats showed significantly higher FBG levels, impaired glucose tolerance, more severe insulin resistance, severe pancreatic damage, and higher levels of dyslipidemia than normal control rats, which indicated the successful establishment of the T2DM model. The insulin resistance model induced by dexamethasone *in vitro* has been widely used. The aqueous extract of *Gynura divaricata* (L.) improving glucose and lipid metabolism was elucidated by establishing an obesity animal model induced by high-fat diet in combination with streptozocin and an insulin-resistant HepG2 cell model induced by dexamethasone (Li J et al., 2018). A beneficial effect of total proanthocyanidins on dysregulated lipid metabolism was demonstrated in high-fat-diet/STZ-induced T2DM mice, and the effects of six flavan-3-ols on adipogenesis and Dex-induced mitochondrial dysfunctions in 3T3-L1 adipocytes were determined to identify the mechanisms (Tie et al., 2020). The HepG2 cells induced with insulin resistance by dexamethasone, together with type 2 diabetes mellitus (T2DM) model rats induced by feeding a high-fat diet for 8 weeks and injecting intraperitoneally with 30 mg/kg STZ, were used to investigate the effects of cajanonic acid A on the regulatory mechanisms of glucose and lipid metabolism (Yang et al., 2018). In other literatures, gastrodin in rat plasma reached the Cmax (13.48 μg/ml) at 70 min after oral administration (40 mg/kg) (Zhang et al., 2008). In this study, the concentrations of gastrodin used *in vitro* were 10 μM (2.86 μg/ml), 30 μM (8.58 μg/ml) and 100 μM (28.6 μg/ml). Therefore, 30 μM Gastrodin treatment *in vitro* was approximately equivalent to oral administration (40 mg/kg).

It has been reported that gastrodin alleviates complications caused by diabetes. Gastrodin attenuates diabetic encephalopathy (Ye et al., 2018). Gastrodin ameliorates the impairment of glucose metabolism and blood flow in orchidectomized (ORX) rats, and gastrodin may be a potential preventive measure for reducing the risk of cardiovascular diseases (Kim et al., 2017). In this study, lowering blood glucose levels and improving glucose tolerance were the primary treatment targets for diabetic patients. After 6 weeks of treatment, compared to the T2DM group, all gastrodin treatments showed a significant decrease in FBG levels. Moreover, the gastrodin treatments (60 and 90 mg/kg) significantly improved glucose tolerance. After 6 weeks of treatment, the hypoglycemic effects of gastrodin were relatively similar to those of metformin. These results suggested that the hypoglycemic activity of gastrodin is reliable in treating T2DM. Insulin-resistant rats also exhibited abnormal lipid metabolism, such as TC, TG, and LDL-c increases and HDL-c decreases. Administration of gastrodin for 6 weeks increased HDL-c and reduced TC, TG, and LDL-c levels, indicating its potential hypolipidemic activity. The effect of gastrodin was similar to that of metformin. Metformin decreases fasting insulin concentration and FBG. TG, TC and LDL were decreased after metformin treatment (Ma et al., 2014).

The occurrence and development of diabetes mellitus is closely associated with insulin resistance, and insulin resistance can lead to multiple organ damage in diabetic conditions. Reducing insulin resistance is an important method to treat T2DM. The insulin sensitivity index (ISI), revealing insulin sensitivity in clinical and animal studies, was significantly decreased in untreated diabetic rats, while gastrodin considerably increased ISI values in T2DM rats, indicating the beneficial actions of gastrodin. Furthermore, gastrodin repaired degeneration and atrophy of islets, and increased islet B-cell expression. Metformin increases insulin sensitivity, but it has no effect in improving islet B-cell function (Ma et al., 2014). Moreover, our findings that gastrodin significantly increased glucose uptake in HepG2 cells also show that gastrodin alleviates insulin resistance.

The regulation of insulin receptor ubiquitination is one of the mechanisms of insulin resistance. Under pathological conditions, insulin receptors can be bound by multiple E3 ubiquitination enzymes and modified by ubiquitination. After ubiquitination of the insulin receptor, it can be further degraded by the proteasome, and the level of the insulin receptor on the cell membrane surface is reduced, resulting in insulin resistance (Nagarajan et al., 2016; Yang et al., 2016).

USP4 is a deubiquitinating enzyme and may deubiquitinate insulin receptors to inhibit their degradation, which maintains the expression level of insulin receptors on the membrane surface and improves insulin resistance. Although there have not been reports that USP4 is directly associated with diabetes, the high expression of USP4 has an ameliorative effect on other metabolic diseases (Zhao et al., 2018). In addition, USP4 plays an important role in the Akt pathway. The deubiquitinating enzyme USP4 modulates AKT regulation of mesothelial-to-mesenchymal transition in peritoneal dialysis (Xiao et al., 2015). The activation of mTORC1 is dictated by a dynamic opposing act between Rheb ubiquitination and deubiquitination that are catalyzed by RNF152 and USP4, respectively (Deng et al., 2019). In this study, treatment of HepG2 cells with Dex upregulated the ubiquitin level of the insulin receptor, but gastrodin reversed this effect. Treatment of HepG2 cells with Dex increased the binding of MARCH1 to insulin receptors, while gastrodin had no effect on the binding of MARCH1 to insulin receptors. Furthermore, gastrodin significantly upregulated the expression of USP4 and increased the binding of USP4 to the insulin receptor.

Considering the importance of the PI3K-Akt pathway in the pathogenesis of T2DM, we detected the effects of gastrodin on the PI3K-Akt pathway to further explore the underlying mechanism of gastrodin in ameliorating insulin resistance. It has been reported that gastrodin activates the PI3K-Akt pathway. Gastrodin improves the symptoms of cardiovascular and cerebrovascular diseases. Gastrodin prevents human umbilical vein endothelial cell injury *via* the Akt pathway (Chen et al., 2021). Gastrodin activates the PI3K/AKT and NF-κB pathways and attenuates hypoxic injury in H9c2 cells by upregulating miR-21 (Xing and Li, 2019). Gastrodin protects against hypoxia/reoxygenation (H/R) injury of myocardial cells in neonatal rats by reducing the level of autophagy through the activation of mTOR signals in the PI3K-Akt pathway (Li X et al., 2018). Gastrodin has a protective effect on nerve cells. The administration of gastrodin provides neuroprotection against early brain injury after experimental subarachnoid hemorrhage (SAH), and gastrodin significantly upregulates the expression of phospho-Akt (Wang et al., 2019). Gastrodin has neuroprotective effects, and activation of the Akt/Nrf2 pathway may play a critical role in the neuroprotective effects of gastrodin (Peng et al., 2015).

In this study, gastrodin upregulated the phosphorylation levels of GATA1 and Akt and stimulated the PI3K/AKT pathway. In addition, compared with the model group, the binding level of GATA1 and the USP4 promoter was significantly increased in the gastrodin group. It has been reported that the activation of the PI3K/AKT pathway induces the phosphorylation of GATA-1 (Zhao et al., 2006). Moreover, administration of the PI3K/AKT signaling pathway inhibitors MK-2206 and LY294002 abolished the beneficial effects of gastrodin. Thus, these results indicate that gastrodin promotes the phosphorylation of GATA1 and upregulate its transcriptional activity *via* the PI3K-Akt pathway, promotes USP4 gene transcription, and upregulates USP4 expression. Upregulation of USP4 reduces the ubiquitination and degradation of insulin receptors, upregulates the level of insulin receptors, and ultimately improves insulin resistance. Gastrodin may ameliorate T2DM through multiple mechanisms. However, these mechanisms have not yet been fully elucidated, so further studies to clarify the mechanism of gastrodin in the treatment of T2DM are needed.

## Data Availability

The original contributions presented in the study are included in the article/Supplementary Material, further inquiries can be directed to the corresponding author.
